# Logic-based modeling and drug repurposing for the prediction of novel therapeutic targets and combination regimens against E2F1-driven melanoma progression

**DOI:** 10.1186/s13065-023-01082-2

**Published:** 2023-11-22

**Authors:** Nivedita Singh, Faiz M Khan, Lakshmi Bala, Julio Vera, Olaf Wolkenhauer, Brigitte Pützer, Stella Logotheti, Shailendra K. Gupta

**Affiliations:** 1 Department of Biochemistry, BBDCODS, BBD University, Lucknow, Uttar Pradesh India; 2 MRC Laboratory for Molecular Cell Biology, University College London, London, UK; 3 Department of Systems Biology and Bioinformatics, University of Rostock, Rostock, Germany; 4 Department of Dermatology, Universitätsklinikum Erlangen and Friedrich-Alexander Universität Erlangen-Nürnberg (FAU), Erlangen, Germany; 5 Leibniz Institute for Food Systems Biology, Technical University of Munich, Munich, Germany; 6 Chhattisgarh Swami Vivekanand Technical University, Bhilai, Chhattisgarh India; 7 Stellenbosch Institute of Advanced Study, Wallenberg Research Centre, Stellenbosch University, Stellenbosch, South Africa; 8 Institute of Experimental Gene Therapy and Cancer Research, Rostock University Medical Center, Rostock, Germany; 9 DNA Damage Laboratory, Physics Department, School of Applied Mathematical and Physical Sciences, National Technical University of Athens (NTUA), Zografou, Athens, Greece; 10 Comprehensive Cancer Center Erlangen-European Metropolitan Area of Nuremberg (CCC ER-EMN), Erlangen, Germany; 11 Deutsches Zentrum Immuntherapie (DZI), Erlangen, Germany

**Keywords:** Melanoma, Network modeling, Perturbation, E2F1, Drug repurposing, Systems pharmacology, Virtual screening, AKT1, MDM2

## Abstract

Melanoma presents increasing prevalence and poor outcomes. Progression to aggressive stages is characterized by overexpression of the transcription factor E2F1 and activation of downstream prometastatic gene regulatory networks (GRNs). Appropriate therapeutic manipulation of the E2F1-governed GRNs holds the potential to prevent metastasis however, these networks entail complex feedback and feedforward regulatory motifs among various regulatory layers, which make it difficult to identify druggable components. To this end, computational approaches such as mathematical modeling and virtual screening are important tools to unveil the dynamics of these signaling networks and identify critical components that could be further explored as therapeutic targets. Herein, we integrated a well-established E2F1-mediated epithelial-mesenchymal transition (EMT) map with transcriptomics data from E2F1-expressing melanoma cells to reconstruct a core regulatory network underlying aggressive melanoma. Using logic-based in silico perturbation experiments of a core regulatory network, we identified that simultaneous perturbation of Protein kinase B (AKT1) and oncoprotein murine double minute 2 (MDM2) drastically reduces EMT in melanoma. Using the structures of the two protein signatures, virtual screening strategies were performed with the FDA-approved drug library. Furthermore, by combining drug repurposing and computer-aided drug design techniques, followed by molecular dynamics simulation analysis, we identified two potent drugs (Tadalafil and Finasteride) that can efficiently inhibit AKT1 and MDM2 proteins. We propose that these two drugs could be considered for the development of therapeutic strategies for the management of aggressive melanoma.

**Graphical abstract:**

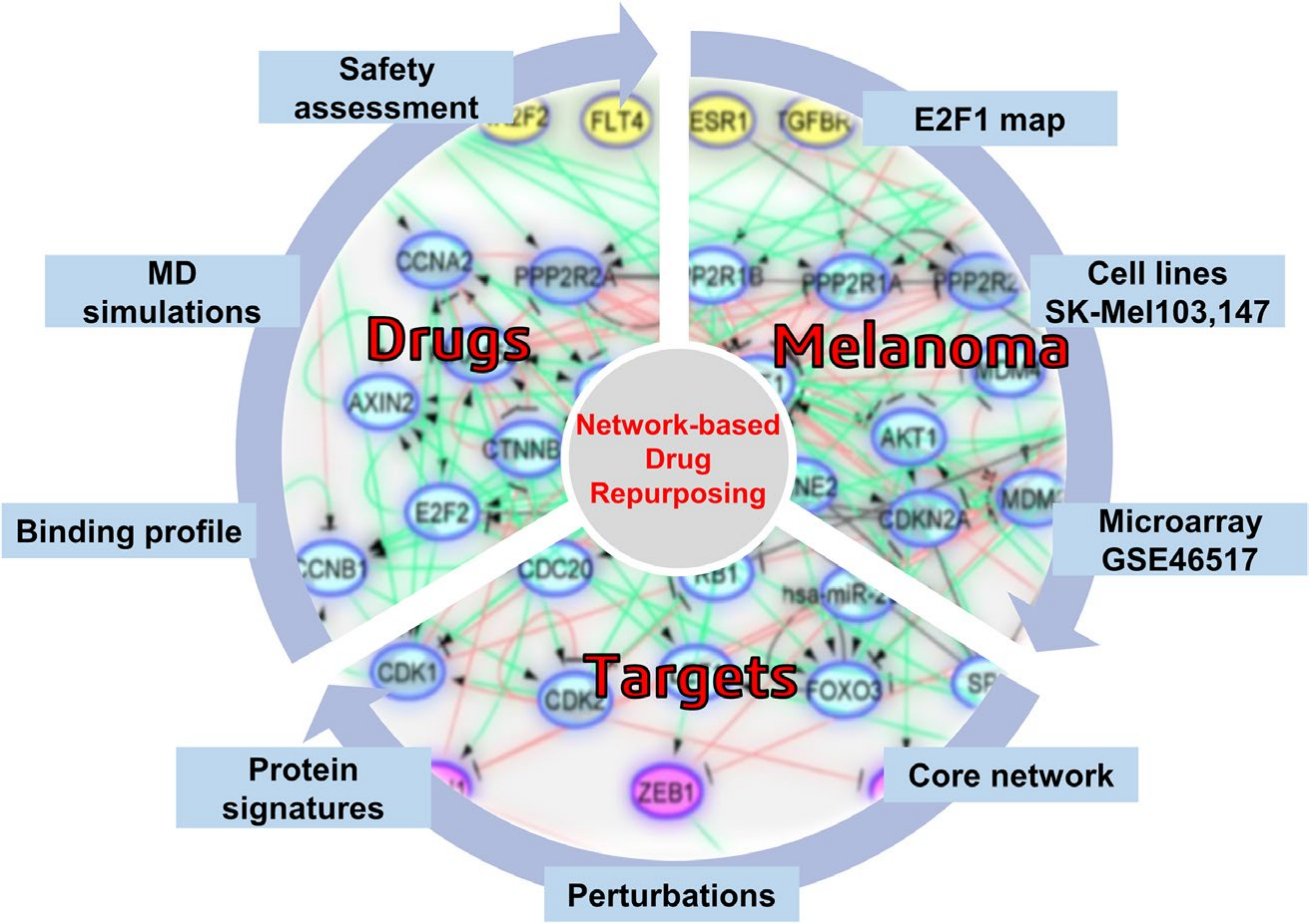

**Supplementary Information:**

The online version contains supplementary material available at 10.1186/s13065-023-01082-2.

## **Introduction**

Cutaneous melanoma arises from melanocytes and represents the deadliest form of skin cancer, with increasing prevalence. Once it becomes metastatic, the prognosis is very unfavourable. Melanoma formation is driven by mutations in the BRAF and NRAS oncogenes [1]. However, these oncogenic aberrations are early events in melanoma genesis that alone do not seem to be sufficient to drive metastasis [2]. Over the past years, we [3–5] and others [6, 7] have demonstrated that in addition to these driver events, melanoma progression is catalyzed by the abundant expression of E2F1, a member of the E2F transcription factor family. Although this transcription factor activates tumor-suppressive pathways at early oncogenesis, upon disease progression unbalanced E2F1 activity is rewired to deregulated cancer networks that underlie hallmarks of metastatic progression such as resistance to apoptosis, chemoresistance [3, 8], neoangiogenesis [9], extravasation [6], EMT [10, 11], metabolic reprogramming [12], and genomic instability [13]. By integrating logic-based network modeling and gene expression profiles of cancer cell lines from E2F1-driven tumors and patient cohorts displaying cancer aggressiveness, we identified tumor-type specific receptor signatures associated with EMT, where the combined action of highly expressed E2F1, TGFBR1, and FGFR1 triggers the most invasive phenotype [10, 14]. Several other protein-coding genes, miRNA genes, and lncRNA genes have been identified as constituents of E2F1-activated prometastatic GRNs [10, 12, 13, 15, 16]. Recurrent structural patterns known as feedforward and feedback regulatory motifs are formed among different regulatory network layers within the E2F1-governed GRNs. They are composed of protein-coding and non-coding RNA genes [11, 15, 16], and are frequently found in cancer networks [17, 18]. These regulatory motifs can cause a wide variety of dynamic behaviors, making them difficult to identify using traditional data mining techniques [19]. Consequently, computational and systems biology-based techniques are needed for the prediction of potential therapeutic targets within the network (more details about regulatory motifs is provided in the additional supp file 3).

Uncovering major epigenetic features and the immune contexture of melanoma has catalyzed the development of anti-melanoma therapies within less than two decades. Until 2004, no systemic therapies for melanoma had been shown to provide a survival benefit. Now, at least four regimens of targeted therapy and three for immunotherapy improve overall survival and disease-free survival, with each modality presenting distinct benefits and limitations. Particularly in 2011, vemurafenib became the first BRAF-targeted therapy approved by the Food and Drug Administration (FDA) for the treatment of melanoma [20]. Unfortunately, despite being impressive and rapid, responses to BRAF inhibitor monotherapy were transient. In most cases, this was due to the development of resistance via reactivation of the mitogen-activated protein kinase (MAPK) pathway. Combined BRAF and MEK inhibition addresses this MAPK-mediated mechanism of resistance and constitutes the current standard-of-care for targeted melanoma therapy. Compared to BRAF inhibitor monotherapy, regimens of BRAF plus MEK inhibitors produce long-lasting disease control and are more tolerable, but a major concern is the resistance that eventually develop, even if it takes some time [21, 22]. Likewise, the treatment response of patients with mutant NRAS-positive metastatic melanoma to MEK inhibitors is transient and short-lived [23]. With the advances in cancer immunotherapy, several next-generation immune-based formulations, such as the checkpoint inhibitors ipilimumab, pembrolizumab, and nivolumab, have received FDA approval for the indication of metastatic melanoma and ensure durable responses. However, they are linked with immune-related toxicities and pose limitations for use in patients with either an overactive (autoimmune disease patients) or a suppressed (organ transplant recipients) immune system [21, 22, 24]. It is therefore essential to develop both, effective and safe strategies, that specifically interfere with the complex melanoma networks. Combining anticancer drugs is currently seen as the approach most likely to overcome single-agent resistance, to produce sustained clinical remissions via multi-targeting effects on distinct mechanisms of action, and to reduce unwanted side-effects by usage of lower drug doses [25, 26]. In fact, the need for combinatory therapies is an inevitable consequence of the evolving nature of tumors. Clonal evolution is particularly active when tumors are under selective pressures due to medical treatments, thereby promoting resistance to therapy. The idea that early administration of combinatorial treatments stands a higher chance of eliminating such clones while their number is extremely low, before acquired resistance is explicitly detected, is supported by the fact that resistant cell clones frequently pre-exist at the beginning of therapy. Simultaneous targeting of the driver oncogenic mutations along with the expected secondary resistance may provide a significant advantage in survival compared with administration at relapse. However, ab initio combination therapies are challenging in the clinical oncology setting because of the narrow therapeutic window between tumor cells and host, which overall limits the number of agents that can be simultaneously tested [26, 27]. With recent advances in high-throughput screening methods, a systematic evaluation of combinations among large collections of chemical compounds in vitro has become feasible. This typically requires large-scale experiments, in which the combinatorial responses are tested in various doses on cancer cell lines or patient-derived cells, resulting in dose-response matrices that capture the measured combination effects for every concentration pair in a particular sample [25, 26]. However, even with modern high throughput instruments, experimental screening of drug combinations can become a herculean task, as the number of conceivable drug combinations increases rapidly with the number of drugs under consideration. In addition, the inherent heterogeneity of cancer cells further challenges the experimental efforts, as the combinations need to be tested in various cell contexts and genomic backgrounds. Hence, computational methods are often recruited to guide the discovery of effective combinations that can be prioritized for further pre-clinical and clinical validations [26, 28].

Herein, aided by in silico workflows, we sought to predict efficient and safe compounds that either alone or in combination prevent melanoma progression by specifically targeting components of the prometastatic E2F1-governed GRNs in melanoma. Using a comprehensive regulatory and functional map of E2F1 in tumor progression and metastasis [10] which contains different types of regulatory factors, including genes, proteins, microRNAs, or complexes, we identified a core regulatory network in melanoma [29, 30]. The core regulatory network was subjected to logic-based modeling for detecting protein signatures which play an important role in interconnecting many of the responsive genes that are typically not identified through gene-based differential expression analysis. Logic-based models uses boolean algebra to present such interconnections and provide robust predictions of emergent behaviours in networks [31]. The subsequent virtual screening, which is a major contributor to computer-aided drug design (CADD) and drug repurposing concept, are increasingly popular techniques that improve the speed and efficiency of the drug discovery process [32, 33], was applied to find FDA-approved drugs against prioritized protein signatures. This combined approach allows us to take advantage of existing safety profiles and established pharmacokinetic properties of approved drugs [34]. Our approach supports the role of AKT1 and MDM2 protein signatures as drivers of EMT in melanoma cancer and suggests that MDM2 plus AKT1 inhibitors could be a promising combination for a novel anti-metastatic regimen in high E2F1-expressing melanoma patients.

## Results

### Establishment of a computational pipeline for the prediction of drug-targetable components of the E2F1-governed prometastatic GRN in melanoma and in silico screening of different inhibitors, alone or in combination

Previously, we have designed a comprehensive regulatory and functional map of E2F1 in tumor progression and metastasis [10] which contains different types of regulatory factors (n = 879) including genes, proteins, microRNAs, or complexes; and interactions (m = 2278) based on information retrieved from published literature and databases. The map was modularized into three E2F1 regulatory compartments such as extra-/intracellular receptor signaling, post-translational modifications, regulators of E2F1 activity; and seven functional compartments including cell cycle, quiescence, DNA repair, metabolism, apoptosis, survival, and angiogenesis/invasion. Using a computational pipeline, we used the map to unravel a tumor type-specific regulatory core and to predict receptor protein signatures in bladder and breast cancer underlying E2F1-mediated EMT transition. The E2F1 map and the previously used workflow [10] were applied to identify a key functional module (core regulatory network) in melanoma. This core regulatory network is composed of regulatory motifs and critical molecular interactions that drive phenotype switching in melanoma. The core regulatory network for melanoma was subjected to our computational pipeline to detect protein signatures that play an important role in interconnecting many of the responsive genes that are typically not identified through gene-based differential expression analysis. The computational pipeline is time-efficient and effective to identify therapeutic targets in a systematic manner [35] as it (i) generates visual interactive networks through provided databases and is not constrained by the lack of quantitative mechanical data [36], (ii) does not depend on negative samples and the three-dimensional structure of target proteins [37], (iii) is useful for multi-target set identification in multi-target drug development [38], and (iv) can be compared with experimental methods that always restrict cellular processes to one element or signaling pathway.

**Fig. 1:**
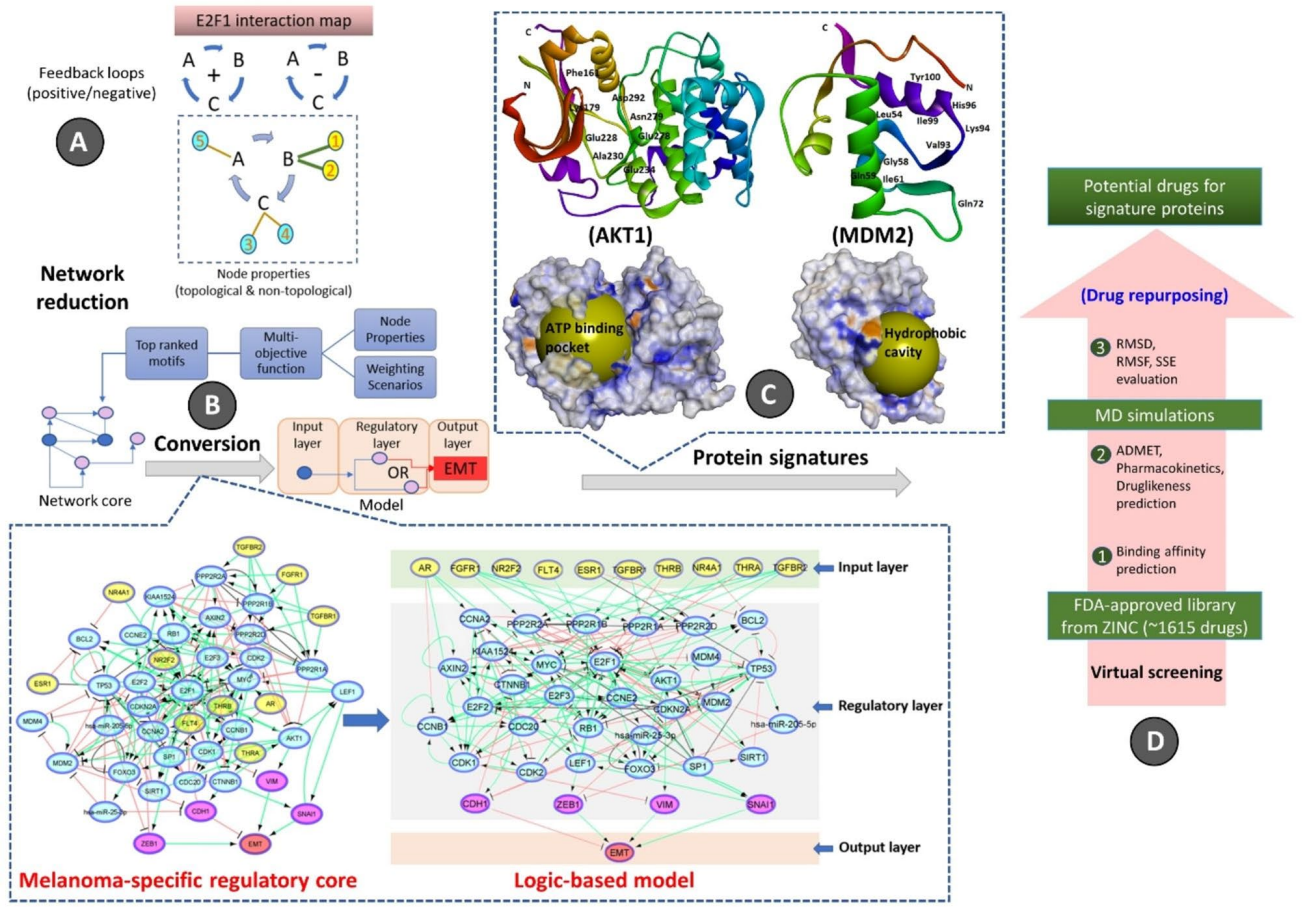
Workflow for the identification and prioritization of therapeutic targets regulating metastatic melanoma phenotypes and virtual screening of repurposed drugs. The overall workflow is divided into four parts. (**A**) The E2F1 interaction map was used to derive positive/negative feedback loops followed by the calculation of node properties, network reduction via a multi-objective function, and subsequently merging the top-ranked motifs to generate a network core. (**B**) Melanoma-specific regulatory core: The constructed core consists of 183 direct interactions (edges) involving 34 core proteins and miRNAs, 10 receptor proteins, and 4 EMT marker proteins. Regulatory directions were retrieved from the E2F1 map as activation(+ 1), inhibition(-1), and unidentified(0). Logic-based model: The model is divided into three layers: the input layer containing receptor molecules (yellow color nodes), the middle layer comprising the regulatory network molecules (cyan color nodes) with known-marker proteins (pink color nodes), and the output layer containing the EMT phenotype (red color node). Green color edges represent activation, red color edges represent inhibition and gray edges represent neutral regulatory relationships among the nodes. (**C**) Two protein signatures (AKT1 and MDM2) were identified through the in silico perturbation experiments on the logic-based model. The functional binding sites of AKT1 and MDM2 are shown in the ribbon model with key amino acid residues participating in the binding pocket formation. At the bottom, the surface models of AKT1 and MDM2 are exposed. In the case of AKT1 (PDB: 3OCB_chainA) the kinase domain showing the ATP binding pocket is identified as the main binding pocket however, in the case of MDM2 (PDB: 3JZK_chain A) the binding site is identified as the main hydrophobic cavity that interacts with p53, displayed in yellow spheres respectively. (**D**) Virtual screening highlights various filtering steps for the identification of potential drug inhibitors from FDA-approved drug library

The proposed computational pipeline (Fig. 1) here includes (i) network-based analysis of topological parameters to characterize the pattern of factors in a networked system, (ii) mapping of the gene expression profiles from melanoma cell lines onto the E2F1 map, (iii) identification of core regulatory network via a multi-objective function to provide motif ranking by user-defined weights in an iterative manner, (iv) boolean modeling of the core regulatory network to analyze and predict the protein signatures linked to aggressiveness in melanoma, (v) structure-based virtual screening and molecular dynamics simulation (MDS) studies to find repurposed drugs against protein signatures that elicit measurable biological responses, and (vi) to predict ADMET behaviors and pharmacokinetic parameters of candidate drugs.

### Identification of the metastatic melanoma-specific core regulatory network

We used our previously published network-based approach to construct a melanoma-specific regulatory core from the comprehensive E2F1 GRN [10]. Here, we utilized the workflow and the E2F1 map to identify key network motifs and critical molecular interactions that drive a highly invasive melanoma cell phenotype. To do this, we have used the data extracted from the E2F1 map and identified important network motifs by calculating of topological and non-topological parameters of each node (Fig. 1A) (additional supp file 1a). The motifs were prioritized using a multi-objective optimization function. For this, weights are assigned to each parameters based on their importance in an iterative and user-defined manner to rank the motifs according to the value of the objective function. The top ten high-scored motifs were selected from each weighting scenario (additional supp file 1a-c). Finally, we merged all the top-ranked motifs to obtain a melanoma-specific regulatory core. We expanded the regulatory core by adding receptor proteins which are the first neighbors of ranked motif nodes in the E2F1 map. We also added four well-known markers CDH1, VIM, ZEB1, and SNAI1 [39, 40] in the core network (additional supp file 1d) to measure the EMT response (Fig. 1B).

### Boolean modeling of the melanoma-specific core regulatory network

**Fig. 2:**
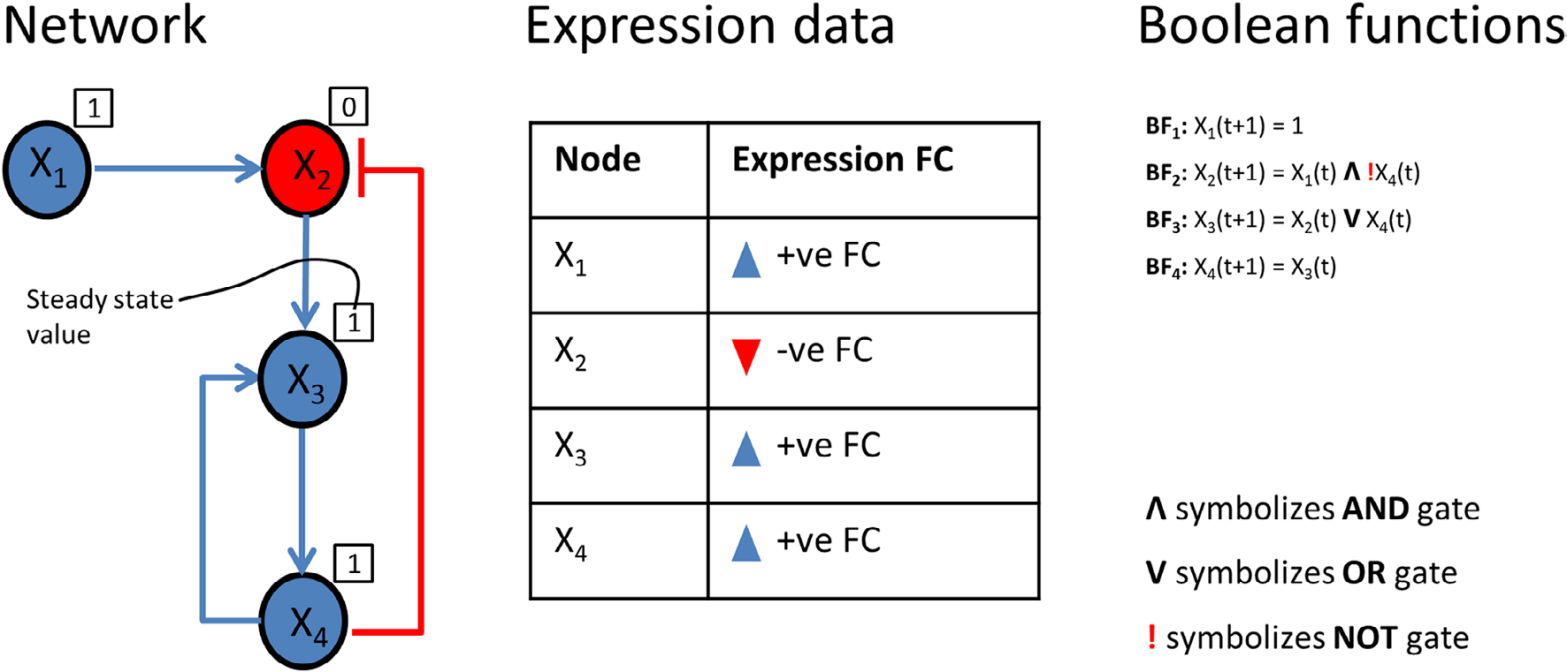
Logic-based representation of the model. The network contains four nodes (X1, X2, X3, and X4). The regulatory relationships are represented by arrows (representing activation) and a t-headed line (representing inhibition). Node X1 is represented as an input of the model because it has no regulator and due to the + ve FC value in the expression table, its state is initialized by ‘1’ (see BF1). Node X2 has two regulators: X1(activator) and X4 (inhibitor). The FC values of both X1 and X4 are + ve but the FC of X2 is –ve indicating the collective effect of both regulators on X2, therefore, it is encoded by the AND gate (see BF2). Node X3 has two regulators: X2 (activator) and X4 (activator). The FC expression values of X2 and X4 are –ve and + ve respectively while the FC of X3 is + ve which indicates the independent effect of regulators on X3, therefore, it is encoded by OR gate (see BF3). Node X4 is activated by only regulator X3 and both of them have + ve FC, therefore, it is encoded as the future state of X4 is dependent on the present state of X3 (see BF4). After initializing the input node, simulation was performed to ensure that the steady-state value of a node (box over the node containing 0 or 1) matched to the FC expression value (red or blue color of the node) i.e., a node with a + ve FC (blue color) is represented by a steady-state value of 1 and node with a –ve FC (red color) is represented by 0

We encoded the core-regulatory network into a boolean model for stimulus-response and perturbation analyses. Stimulus-response analysis was used to identify the effect of up/down expressed receptors on the EMT phenotype, and perturbation analysis predicted a potential drug target that can bring the phenotype to the lowest possible level. In the boolean model, the state of a node is represented with two possible conditions i.e., 0 (OFF, inactive) or 1 (ON, active) [41]. The regulatory relationships between upstream nodes (i.e., sources) to downstream nodes (i.e., targets) are encoded into boolean functions using logical gates ‘NOT’, ‘OR’, and ‘AND’ [42] (Fig. 2). ‘NOT’ operator encodes inhibitory relation. ‘OR’ operator is used to express the relationship when a target is regulated by multiple regulators independently, i.e., the target will be active if any one of the regulators is active. ‘AND’ operator encodes the collective effect of multiple regulators on a target. Further, we calibrated the boolean functions with fold-change (FC) expression data [41] of the publicly available dataset GSE46517 [43] from Gene Expression Omnibus (GEO). To evaluate the input-output behavior, we divided the model into three layers: (1) the input layer, containing receptor molecules, (2) the regulatory layer, comprising nodes constituting a core-regulatory network, and (3) the output layer, including EMT phenotype (Fig. 1B). The input layer was initialized with an FC expression profile i.e., a node with negative FC was represented by a state 0, and a node with positive FC was represented by a state 1 (Fig. 2).

### In silico perturbation simulations using boolean modeling

The model was simulated with initial values derived from the expression profile of input nodes and confirmed that the logical state of nodes in a regulatory layer represents the data (for details https://github.com/nivisingh22/Melonoma_core_model). The EMT phenotype was regulated by nodes ZEB1, CDH1, VIM, and SNAI1 [11], and represented by 5 ordinal levels ranging from 0 (minimum) to 4 (maximum):

**EMT = ZEB1 + NOT (CDH1) + VIM + SNAI1**

For the initial condition, model simulations result in EMT of level 3, where ZEB1 and SNAI1 are active and CDH1 is inactive (see Table 1a). Further, we performed perturbation analysis of all nodes (except ZEB1, CDH1, VIM, and SNAI1) in the regulatory layer of the model to bring EMT from level 3 to a minimum level. We identified that for a single perturbation (in this case inhibition) of MDM2 or MIR25, EMT can be reduced to level 1 (see Table 1b). CDH1 is activated upon inhibition of MDM2 which inhibits EMT as well as inhibits CTNNB1 which subsequently inhibits SNAI1 [44, 45] to further reduce EMT. A similar effect was observed upon inhibition of MIR25 [46, 47]. On the other hand, a single perturbation (in this case activation) of AKT1 can increase the EMT to the highest level 4.

**Table 1:** Stimulus-response and perturbation simulations. The underlined perturbed nodes have the most effect on the EMT phenotype

AR	ESR1	FGFR1	FLT4	NR2F2	NR4A1	TGFBR1	TGFBR2	THRA	THRB	MDM2	MIR25	AKT1	ZEB1	CDH1	VIM	SNAI1	EMT
**(a) Stimulus-response analysis for the initial condition.** **(Model simulation results of initial condition which results in higher EMT level.)**																	
NaN	1	1	NaN	1	1	1	NaN	0	NaN	1	1	0	1	0	0	1	3
**(b) Single perturbations analysis (inhibition of MDM2, MIR25 and activation of AKT1) for EMT level of 3.** **(Single perturbation by inhibiting MDM2 or MIR25 can bring EMT from level 3 to 1, while upregulating AKT1 resulted in EMT level to 4.)**																	
NaN	1	1	NaN	1	1	1	NaN	0	NaN	**0**	1	0	1	1	0	0	1
NaN	1	1	NaN	1	1	1	NaN	0	NaN	1	**0**	0	1	1	0	0	1
NaN	1	1	NaN	1	1	1	NaN	0	NaN	1	1	**1**	1	0	1	1	4

Using CellNetAnalyzer, we conducted a systematic evaluation of the network response if the model is confronted with failures. By interpreting a failure as something that results from either intracellular or external fluctuations in the cell e.g. a mutation event. We found that the model is robust against single failure except for AKT1 and MDM2, in the regulation of EMT phenotype. In other words, the network is robust against numerous perturbations, and only the AKT1 and MDM2 have a profound effect on the network dynamics, which is the property of a scale-free network (see details of ‘initial conditions’ and ‘output predictions’ in the online link of the model).

### Assessment of protein signatures identified through boolean modeling

**Fig. 3:**
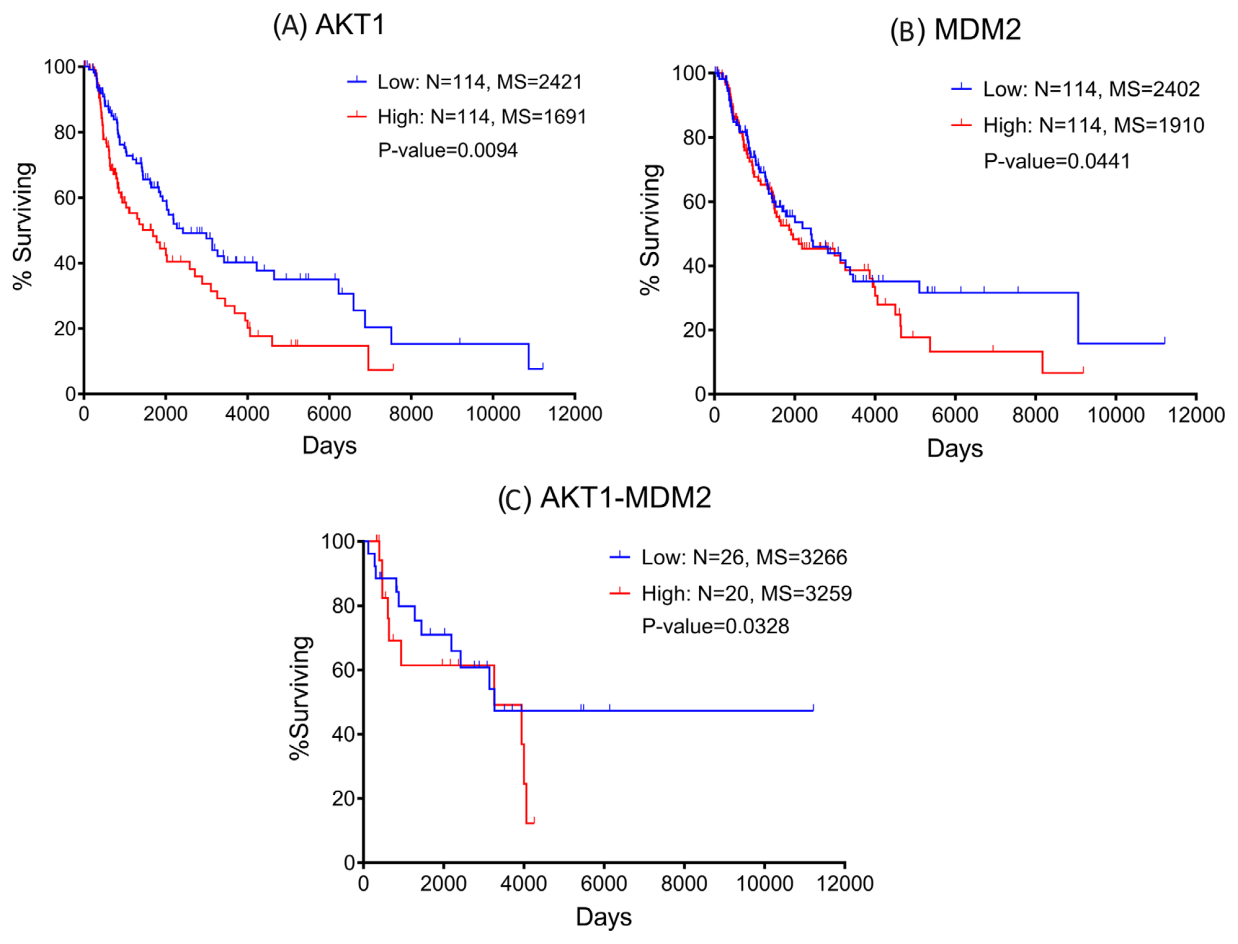
Kaplan-Meier plots suggest that patients with high expression of (**A**) AKT1; (**B**) MDM2; and (**C**) high expression of AKT1 and MDM2 together have the worst clinical outcomes

Our boolean model simulations suggested two key proteins AKT1 and MDM2 that upon inhibition can bring the EMT from level 3 to 1. Interestingly, AKT1 directly activates the VIM, a key marker for EMT. AKT1 also activates MDM2 which interacts with p53 to regulate the immune axis in metastatic melanoma. MDM2 also indirectly activates the EMT by downregulating another hallmark protein CDH1. We investigated the expression profiles of AKT1 and MDM2 and their impact on melanoma patient survival using the Kaplan-Meier curve (Fig. 3) using the TCGA melanoma SKCM dataset. We found that higher expression of both AKT1 and MDM2 resulted in poor patient survival. These observations also confirm that the boolean model simulation was successful in predicting potential proteins that may be targeted for the treatment of metastatic melanoma.

### Screening of FDA-approved drugs to block protein signatures

To identify drugs that are most likely to bind to AKT1 and MDM2 protein signatures, molecular docking was performed with the FDA-approved drug library from the ZINC database (additional supp file 2b-c). The information about the active sites of proteins is retrieved from the literature and PDB database. More specifically, for AKT1 we performed screening against the kinase domain (150–408) which was previously selected to identify ATP-competitive inhibitors [48, 49] (Fig. 1C). For MDM2, many recent studies indicate that its overexpression and subsequent deactivation of p53 result in failure of apoptosis and cancer cell survival [50–52]. We investigated the p53–Mdm2 interaction surface which is ~ 700 Å2. This druggable pocket of MDM2 where p53 binds provides a great opportunity for compound inhibitors to disrupt p53–MDM2 interaction [53] (Fig. 1C).

**Fig. 4:**
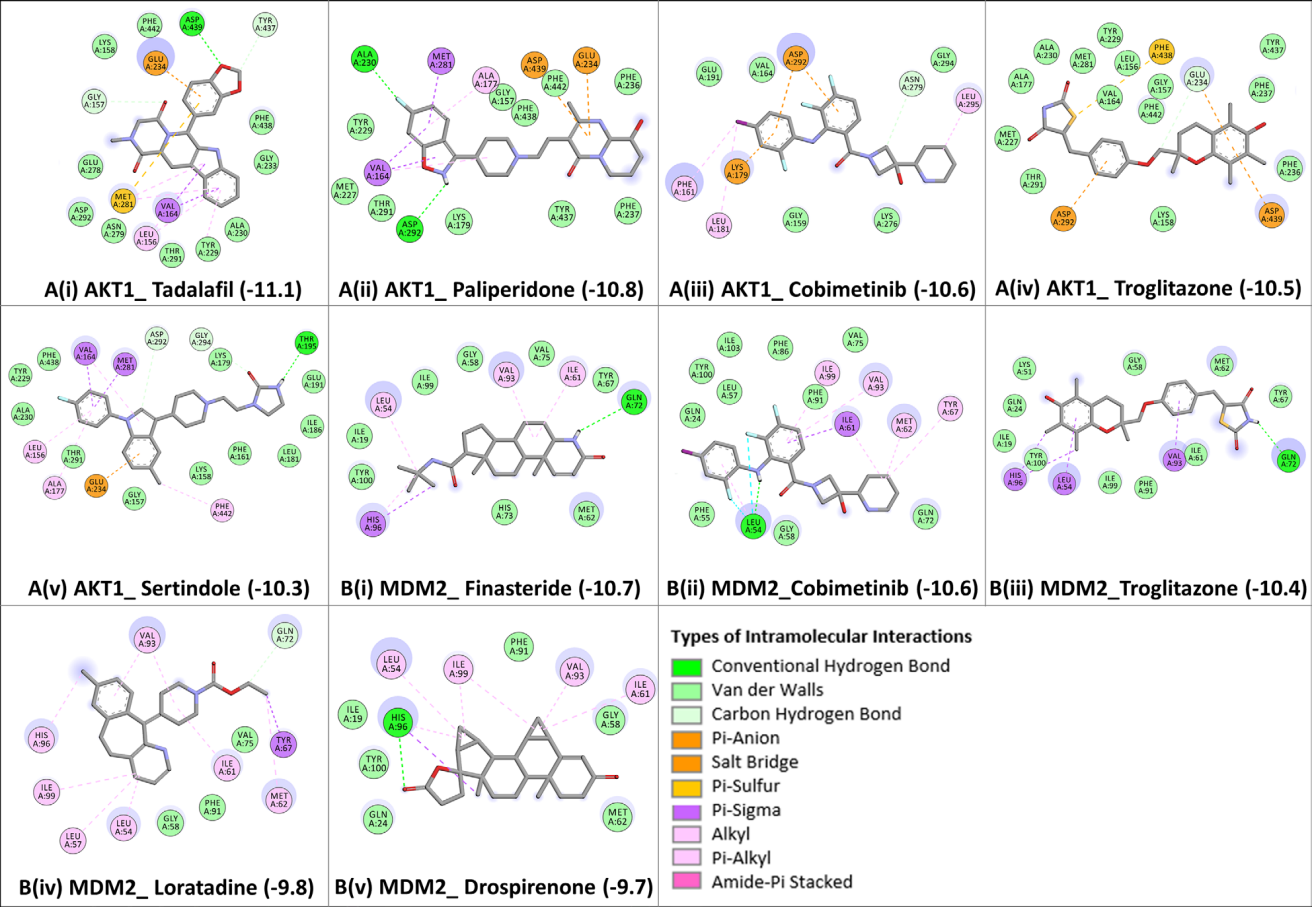
2D interaction diagrams showing the binding profile of both the protein signatures **A(i-v)** AKT1 and **B(i-v)** MDM2 with their top five screened candidate drugs: Tadalafil, Paliperidone, Sertindole, Troglitazone, Cobimetinib, Drospirenone, Finasteride, and Loratadine. The binding affinities are measured in kcal/mol (given in brackets). Intramolecular interactions are depicted as colored dashed lines between protein residues and drug atoms. The solvent-accessible surface of an interacting residue is represented by a blue halo around the residue

From the achieved docked poses for each ligand-protein complex, we selected the pose with the lowest energy value (out of 9 conformations) and compared it with the crystallographic pose, RMSD ≤ 2.0 Å. Further, the best predicted binding mode and the corresponding binding affinity (in kcal/mol) are selected for each complex. In case of AKT1 Fig. 4A (i-v), docking analyses revealed that the candidate drugs are packed against the residues LEU156, GLY157, PHE161, VAL164, ALA177, LYS179, GLU191, HIS194, GLU198, TYR229, ALA230, GLU234, ASP274, ASN279, MET281, ASP292, GLY294, LEU295, TYR437, PHE438, ASP439, and PHE442 and was stabilized by the hydrogen bonds, electrostatic, hydrophobic, and van der walls interactions. The best binding affinity of AKT1 is obtained with Tadalafil (-11.1 kcal/mol), followed by Paliperidone (-10.8 kcal/mol), Cobimetinib (-10.6 kcal/mol), Troglitazone (-10.5 kcal/mol), and Sertindole (-10.3 kcal/mol). The binding affinities and the number of interactions of these candidate drugs towards the ATP binding pocket of AKT1 are comparable to ATP competitive inhibitors [48]. Particularly, GLU234 of the protein backbone is necessary for the AKT1 biological activity. Secondly, the electrostatic interactions and hydrogen bonds to ASP292 in AKT1 are critical because this position is typically occupied by a divalent cation (Mg2+) bound to ATP [54]. Both of the residues (GLU234 and ASP292) of AKT1 were found to bind with previously known ATP-competitive kinase inhibitors [54, 55] and also found with our candidate drugs. Other active site residues that could be seen in binding were ALA230, GLU228, GLU278, ASN279, PHE161, and LYS179.

Tadalafil is a US FDA-approved drug for the treatment of pulmonary arterial hypertension [56]. The drug shows biologic activity in human melanoma and pilot trial studies reported improved clinical outcomes in patients with head and neck cancer (HNSCC) and metastatic melanoma [57, 58]. Furthermore, Tadalafil indicated immune regulatory and antitumor therapeutic effects in hepatocellular carcinoma [59]. The next drug Paliperidone is an antipsychotic drug that reportedly inhibits glioblastoma growth in mice [60]. However, existing evidence is conflicting with its role as the drug increases prolactin levels, which might increase the risk of breast cancer [61]. Further in the list, Cobimetinib/GDC-0973 is FDA-approved for the treatment of patients with metastatic melanoma with a BRAF V600E or V600K mutation, in combination with vemurafenib [62]. Also, in combination with (i) chemotherapy and (ii) Niraparib, with or without atezolizumab are used as a treatment for patients with breast cancer and with advanced platinum-sensitive ovarian cancer (red) respectively [63]. The next drug hit is Troglitazone, which is a type II diabetic drug and, in clinical trials used in combination with lovastatin and their cotreatment was found to induce cell cycle arrest at the G0/G1 phase in Anaplastic thyroid cancer [64]. On the contrary, there have been concerns as it has detrimental side effects such as causing liver toxicity [65]. The last drug hit was Sertindole which exhibits antiproliferative activities in breast cancer with a potential application for the treatment of breast-to-brain metastases [66] and by inhibiting the STAT3 signaling pathway in human gastric cancer cells [67].

In the case of MDM2 Fig. 4B (i-v), the molecular docking was performed into lining residues of this pocket containing amino acids (LEU54, LEU57, ILE61, MET62, TYR67, GLN72, VAL75, PHE86, PHE91, VAL93, HIS96, ILE99, TYE100, and ILE101). These residues form a hydrophobic cavity on the MDM2 protein structure and are potentially occupied by known inhibitors [68, 69]. The best binding affinity of MDM2 is obtained with Finasteride (-10.7 kcal/mol), followed by Cobimetinib (-10.6 kcal/mol), Troglitazone (-10.4 kcal/mol), Loratadine (-9.8 kcal/mol), and Drospirenone (-9.7 kcal/mol). All top five candidates exhibit convincing binding mode into the druggable pocket of MDM2, and specifically hydrophobic interactions with key residues (VAL93 and LEU54) of MDM2 are obtained in all drug hits. Other important interface residues could be seen in binding were LEU57, ILE61, MET62, TYR67, GLN72, VAL75, PHE86, PHE91, HIS96, ILE99, TYE100, and ILE101. The binding affinity of Finasteride with MDM2 is in a similar range with Cobimetinib and Troglitazone; however, binding affinities of Loratadine and Drospirenone are comparatively lower. Finasteride/ Proscar is used for the treatment of alopecia and prostate cancer. In melanoma, the protective effect of finasteride on melanogenesis via downregulation of tyrosinase, TRP-1, MITF, and ACs expression has been demonstrated [70], further in vivo animal experiments are required for confirmation. Another study similar to this reported that Finasteride declines the risk of melanoma following prostate cancer [71]. The effects of Cobimetinib and Troglitazone were described above. The next drug hit is Loratadine which is routinely given to cancer patients in combination with other drugs and substantially improved survival in both breast cancer and cutaneous malignant melanoma [72]. In some instances, Loratadine has been linked to clinically apparent acute liver injury [73]. The use of Drospirenone in birth control pills and causing severe blood clots (thromboembolism) in women is conflicting [74]. Additionally, long-term use may increase the risk of breast cancer [75].

### ADMET profile of top candidate repurposable drugs

**Fig. 5:**
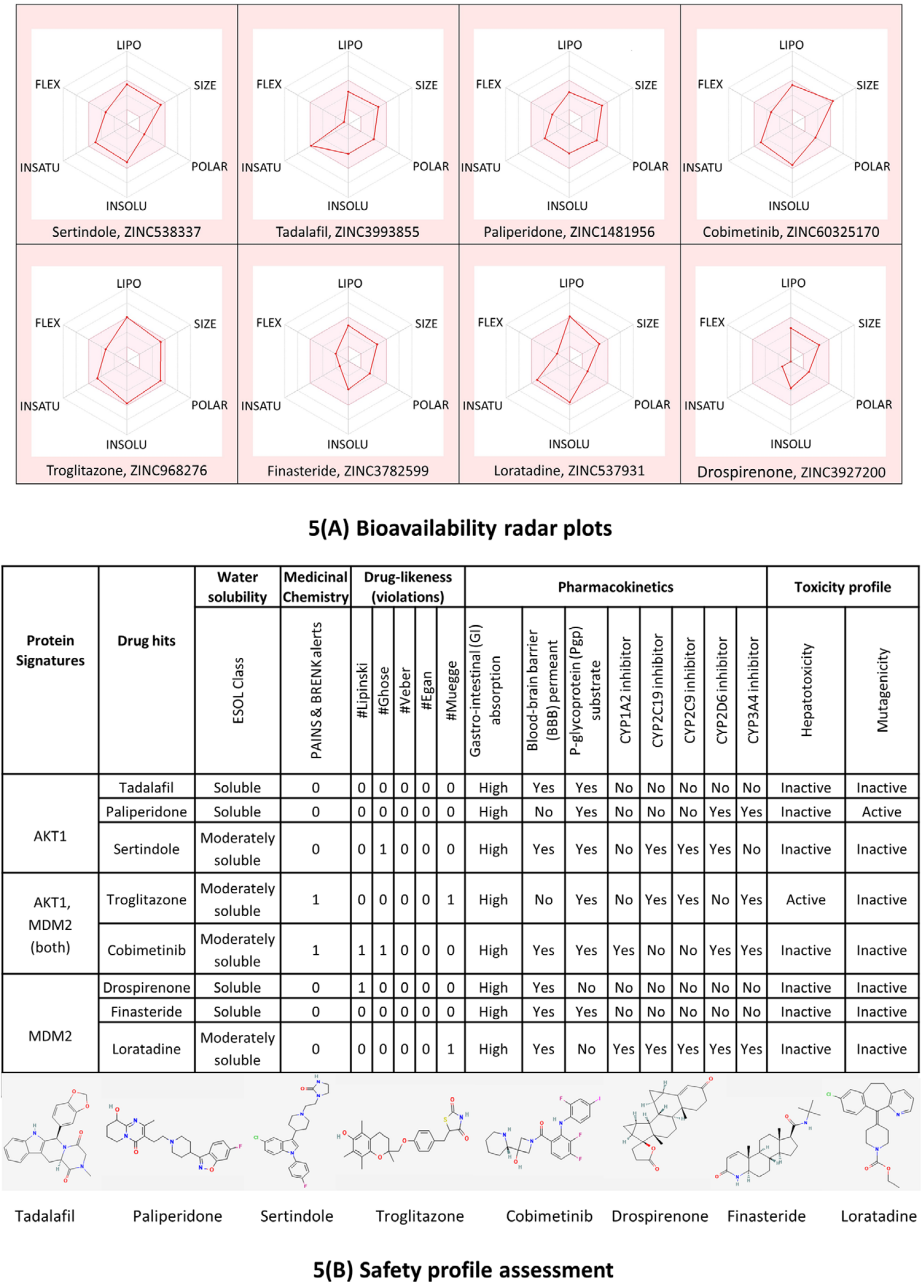
Radar plots: **5(A)** Top screened candidate drugs for oral bioavailability based on six physicochemical properties LIPO (lipophilicity), SIZE (molecular weight), POLAR (topological polar surface area), INSOLU (insolubility), INSATU (in-saturation), and FLEX (flexibility). The pink-colored area represents the ideal range for each property i.e. XLOGP3 (− 0.7 and + 5.0), MW (150 and 500 g/mol), TPSA (20 and 130 Å2), Log S (< 6), Fraction Csp3 (< 1), and Rotatable bonds (< 9), respectively. **5(B)** Prediction of water solubility, medicinal chemistry, drug-likeness, pharmacokinetic, and toxicity profile of top screened candidate drugs

The bioavailability radar plots in Fig. 5(A) show that the hits are falling entirely within the physicochemical range on each axis and hence seem to fit into the bioavailability criteria. The pharmacokinetic profile in Fig. 5(B) shows that all the top candidate drugs have high gastrointestinal (GI) adsorption. Except for Paliperidone and Troglitazone, all the top-hit drugs cross the blood-brain barrier (BBB) and can impair tumor development in brain metastasis. Three drug hits Tadalafil, Paliperidone, and Finasteride showed no violations for Lipinski, Ghose, Veber, Egan, and Muegge rules which suggests that these drugs are likely orally active drugs. Also, no alerts for PAINS and Brenk filters provide information that Tadalafil, Paliperidone, and Finasteride drugs didn’t contain potentially problematic fragments which are toxic or metabolically unstable. It is also essential to predict the interaction of drug hits with cytochromes P450 (CYP) and P-glycoprotein (P-gp) as these are key players in drug elimination through metabolic biotransformation [76]. Tadalafil, Paliperidone, and Finasteride all three are substrates of P-gp [77–79]; and particularly, Tadalafil and Finasteride are not expected to cause unwanted adverse effects due to the lower clearance or accumulation of the drug metabolized by CYP450 isoforms (CYP1A2, CYP2C19, CYP2C9, CYP2D6, CYP3A4).

Overall, the results of molecular docking and ADMET profile suggested two potential strong-binding drug candidates (Tadalafil and Finasteride) of protein signatures (AKT1 and MDM2, respectively), shown to be orally bioavailable, non-toxic, and have good absorption and medicinal properties.

### Molecular dynamics simulation (MDS) and docking validation

Since docking gives a static view of the binding interaction of compound hits into the active site of protein signatures, MDS gives a more clear idea about the physical movements of atoms and molecules with time by integration of Newton’s equation of motion [80]. Therefore, the two docked complexes (AKT1_Tadalafil and MDM2_Finasteride), with the highest predictive binding affinity of -11.1 and − 10.7 kcal/mol, respectively, along with safe therapeutic properties, were used for molecular dynamics study.

**Fig. 6:**
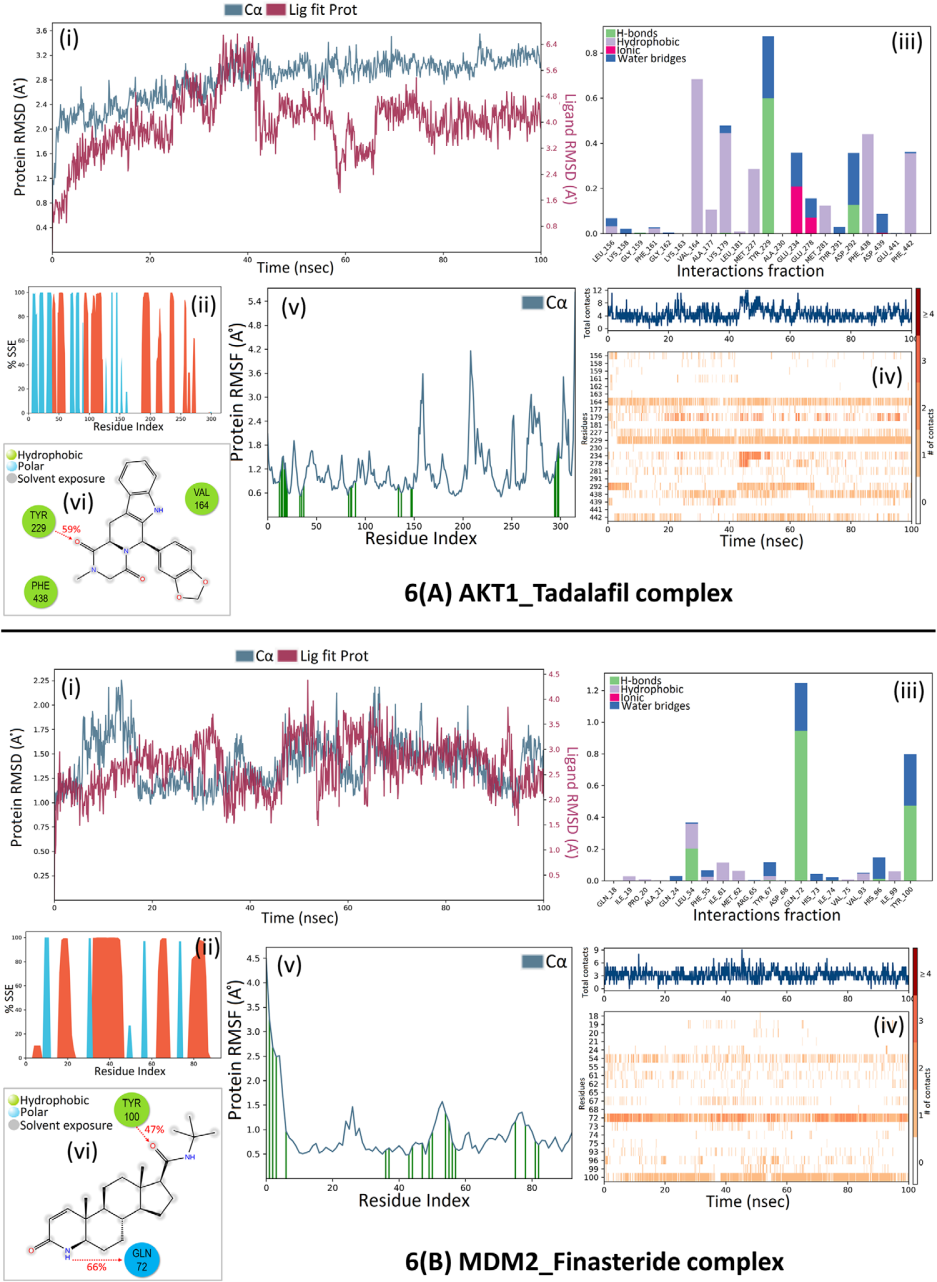
MDS analysis of protein-ligand complex. **6(A)** Simulation results for AKT1_Tadalafil complex and **6(B)** Simulation results for MDM2_Finasteride complex. The graphs show (**i**) PL-RMSD of simulated C-alpha atoms of protein in complex with inhibitor during 100 ns MD simulation. The X-axis shows the variation of protein RMSD through time and the Y-axis shows the variation of protein RMSD through time. (**ii**) P-%SSE histogram showing protein secondary structure element distribution by residue index throughout the protein structure complexed with the ligand. Red columns indicate the alpha helices and blue columns indicate the beta-strands. (**iii**) PL-Contacts histogram showing four types of protein interactions (H-bonds, hydrophobic, ionic, and water bridges) with the ligand throughout the simulation. The stacked bar is normalized over the course of the trajectory. (**iv**) Timeline representation of the interactions and contacts. The top panel shows the total number of specific protein contacts with the ligand and the below panel shows the specific residues which interact with the ligand in each trajectory frame (dense areas represent more than one contact with the ligand). (**v**) P-RMSF of simulated protein in complex with inhibitor during 100 ns MDS. (**vi**) Schematic 2D diagram of ligand atom interactions with the protein residues (interactions that occur more than 30% of the simulation time in the selected trajectory from 0 to 100 ns are only shown)

A 100 ns molecular dynamic simulation was performed to understand the molecular insights involved in the binding of Tadalafil in the active pocket of AKT1. The trajectory analysis (Fig. 6A, (i) PL-RMSD) graph shows that the Ca atoms fluctuated in the range of 2.00–3.50 Å and stabilized after 80 ns of simulation with an RMSD value of 3.0 Å. Higher fluctuation (up to 3.50 Å) in RMSD was observed at 88 ns for the protein. The AKT1 backbone Cα experiences a gradual increase in deviation for the entirety of the simulation period. Also, Tadalafil in complex with AKT1 undergoes a gradual increase in deviation up to 40 ns and then decreases with minor deviations for a period of 40–65 ns; and further maintained a deviation of ~ 2.20 Å for the remainder of the simulation period. The histogram (Fig. 6A, (ii) P-%SSE) shows the secondary structure element (SSE) distribution for each trajectory frame over the course of the simulation. The percentages of the helix, strand, and overall secondary structure elements were found to be 25.06%, 14.63%, and 39.68% respectively. A total of 22 ligand contacts were formed with amino acids of protein (Fig. 6A, (iii) PL-Contacts), from Leu156, Lys158, Gly159, Phe161 to Val164, Ala177, Lys179, Leu181, Met227, Tyr229, Ala230, Glu234, Glu278, Met281, Thr291, Asp292, Phe438, Asp439, Glu441, and Phe442. Fig. 6A, (iv) Timeline graph represents that the ligand is stabilized by forming a majority of hydrophobic interactions with residues Leu156, Phe161, Val164, Ala177, Lys179, Leu181, Met227, met281, Phe438, and Phe442 with 1–68% of simulation time. Hydrogen bonds are formed with residues Gly159, Lys179, Tyr229, and Asp292 throughout 1–60% of the simulation trajectory. The RMSF value of the protein is coupled to the ligand in Fig. 6A, (v) P-RMSF graph of simulated protein AKT1 for 100 ns, with X-axis measuring the average deviation of all protein residues (from 144 to 478) over time and Y-axis indicates RMSF (Å) values. The residues with higher peaks fluctuate the most during the simulation as determined by MD trajectories. Green-colored vertical bars indicate AKT1 residues that interact with Tadalafil and correspondingly, low RMSF values indicate the stability of the binding. Fig. 6A, (vi) 2D-trajectory interaction diagram depicts that the hydrogen bond formed by the docking pose with Tyr229 is preserved in the MD trajectory pose with 59% of the total simulation time.

Further, again a 100 ns molecular dynamic simulation was performed to understand the molecular insights involved in the binding of Finasteride in the p53-MDM2 interaction surface. Fig. 6B, (i) PL-RMSD graph shows that the Ca atoms fluctuated in the range of 1.00–2.25 Å and finally stabilized after 65 ns of simulation with an RMSD value of 1.5 Å. Higher fluctuation (up to 2.25 Å) in RMSD was observed at 15 ns. A slight divergence can be seen towards the end of the simulation around 92 ns. Since, the fluctuation lies under the permissible range of 1–3 Å, hence, can be considered as non-significant. The RMSD plot of Finasteride and MDM2 backbone were lying over each other. Hence, the formation of a stable complex can be inferred. Fig. 6B, (ii) P-%SSE histogram shows the percentages of helix, strand, and the overall secondary structure elements to be 36.11%, 10.12%, and 46.24% respectively. Fig. 6B, (iii) PL-Contacts histogram shows that a total of 20 ligand contacts were formed with amino acids of protein, from Gln18 to Ala21, Gln24, Leu54, Phe55, Ile61, Met62, Arg65, Tyr67, Asp68, Gln72 to Val75, Val93, His96, Ile99, and Tyr100. Fig. 6B, (iv) Timeline representation suggested that the ligand is stabilized by forming Hydrogen bonds with Leu54, Gln72, His96, and Tyr100 with 1-95% of simulation time. Hydrophobic interactions were formed with residues Ile19, Pro20, Leu54, Phe55, Ile61, Met62, Tyr67, Val75, Val93, and Ile99 over the course of 1-36% simulation trajectory. Fig. 6B, (v) P-RMSF graph of simulated protein MDM2 for 100 ns, with the X-axis measuring the average deviation of all protein residues (from 18 to 110) over time and the Y-axis indicating fluctuation RMSF (Å) values. More fluctuations were seen at the C-terminal of the protein during simulation which indicates greater flexibility of the residues while other parts of the protein are more rigid and fluctuate less. Protein residues that interact with the Finasteride are marked with green colored vertical bars. Fig. 6B, (vi) 2D-trajectory interaction diagram depicts that the hydrogen bonds formed by the docking pose with Tyr100 and Gln72 were preserved in the MD trajectory pose with 47% and 66% of total simulation time respectively. The RMSF value of the protein-coupled to the ligand.

## Discussion

A common approach to anticancer drug development has been based on a workflow, whereby molecules that are designed from scratch, to specifically interfere with a certain pathway, are anticipated to target and eradicate tumors in a highly selective manner, analogous to the “lock-and-key” specificity, hence maximizing efficacy and minimizing side effects [81]. Despite their promising results in the preclinical setting, the majority of innovative drugs are proven insufficient or suboptimal when administered in clinical patients, thereby leading to unacceptably low success rates in clinical trials [82, 83]. The high failure rate of this approach is the consequence of several unpredictable parameters, mainly: (a) the individual genetic background of cancer patients, which limits the therapeutic benefits only to specific patient subpopulations and necessitates treatment personalization [21]; (b) the fact that cancer-related genes are highly interconnected and regulate each other through complex loops from different pathways [84]; (c) the inherent ability of tumors to adapt and evolve, which catalyzes acquisition of resistance to therapies, especially monotherapies [27]. To address these challenges, computational methodologies including, but not limited to, algorithms and machine learning tools, are now being increasingly integrated in drug discovery programs. For example, computational approaches that ‘dock’ small molecules into the structures of macromolecular targets and ‘score’ their potential complementarity to binding sites are widely used in hit identification and lead optimization and are currently reforming the pharmacopeia landscape [85]. This approach allows for fast and comprehensive screening of the efficacy and safety profiles of a high number of leads, in the context of a particular cancer type. Prioritization of the top-resulting leads or combinations thereof could subsequently facilitate a faster introduction to clinical trials and significantly reduce the costs for drug development.

Having in mind that metastasis is linked with the activation of E2F1-governed GRNs, we applied a transcriptomics-aided bioinformatics workflow, followed by virtual drug screening to comprehensively characterize novel therapeutic targets in melanoma and predict their corresponding drug inhibitors. Due to the documented ability of targeted drugs to show superior safety and efficacy in combination schemes [21], we were particularly interested in drugs that can perturb these prometastatic GRNs when used simultaneously. Using a well-established E2F1 map [10], we derived a set of three-node FBLs (n = 444) and used a ranking scheme that applies a weighted multi-objective function integrating topological and non-topological properties of each node. In topological properties, the degree of a node (i.e. number of edges connected to the node) is a crucial aspect because it affects how networks are organized and how molecules are connected [86]. A node with a high degree is more approachable, has greater access to resources within the network, and can efficiently spread information throughout the network, especially to less connected nodes [87]. Betweenness centrality is another important topological property for determining how much influence a node has on the information flow in a network. It specifically looks for bridge nodes that link one area of a network to another. For example, Cancer-associated proteins have large betweenness centrality as they control the communication between different components of a network [88]. The full results of the network analysis and topological properties are presented in additional supp file 3.

Among non-topological properties, we have calculated the involvement of the motif constituents in the disease pathway, the gene prioritization score, and the average Log2 fold change for each motif based on the change in expression values of each node from non-invasive to invasive phenotypes derived from in vitro experiments. Since the network was originally constructed around E2F1, the topological properties for some nodes are expected to be higher than other nodes. Therefore, to give equal importance to all nodes, we used different weighting scenarios in the multi-objective optimization function to avoid biases and ranked motifs accordingly. The top-ranked motifs are merged to understand their combined effect on the regulation of EMT in melanoma. We further expanded the regulatory core network by adding receptor proteins the first neighbors of the ranked motif nodes and four marker proteins and their direct connections from the E2F1 map. Receptor proteins work as determinative factors and marker proteins are required to measure the EMT response. We developed a three-layered logic-based model of the regulatory core consisting of an input layer, a regulatory layer, and an output layer. We analyzed the regulatory core by using boolean logic for the input and regulatory layers, and multi-valued logic for the output layer which allows us to assess the combined effect of various network components on the EMT phenotype. Our model simulations identified two protein signatures AKT1 and MDM2 as potential drivers of EMT in melanoma. Further virtual screening of FDA-approved drugs was employed and after binding affinity analysis top five candidate drugs selected for both the proteins AKT1 and MDM2, were evaluated for their safety profile. The candidate drugs with safe therapeutic properties and the least binding affinity with the signatures were subjected to MD simulations to check the conformational stability of the complex and dynamics/flexibility of the protein at 100 ns. The trajectory analysis confirms that the candidate drugs (Tadalafil and Finasteride) stabilized in the active pocket of protein signatures (AKT1 and MDM2) over the course of the simulation (at 80 ns and 65 ns respectively). Analysis of PL-contacts histogram and 2D-trajectory diagrams indicate that the hydrogen bonds formed by the residues TYR229 (in AKT1) and, TYR100 & GLN272 (in MDM2) are critically important residues and observed to play a predominant role in drug binding, thus contributing to the high stability of the complexes and could be further explored for *in-vitro* or *in-vivo* studies.

AKT activation has been shown to be a strong marker of poor prognosis in patient melanoma samples [89]. The AKT family has three isoforms, AKT1, AKT2, and AKT3, which are highly homologous [90]. However, isoform selectivity has been uncovered in the targets and overall function of each isoform, especially regarding cancer. Each AKT isoform has been found to be upregulated in different cancers, with varying impacts on tumor cell proliferation, survival, and metabolism. In melanoma, AKT1 and AKT2 activation are more commonly found in BRAF-mutant tumors, while AKT3 hyperactivity is more common in BRAF wild-type melanomas [91]. Moreover, both AKT1 and AKT2 have been implicated in melanoma metastasis [92, 93]. Importantly, AKT signaling has been connected to senescence, and specifically AKT1 isoform-specific inhibition has been suggested as a novel therapeutic target in melanoma [91]. In agreement with these studies, our analysis highlighted inhibition of AKT1 as an attractive strategy for preventing EMT-driven metastatic progression of melanomas with high-E2F1 content.

In addition, MDM2 was shown to be abnormally upregulated leading to enhanced degradation and reduction of p53 activity in some tumors [94]. Therefore, targeting the MDM2–p53 interaction represents an attractive therapeutic strategy for the reactivation of p53 in cancers with wild-type or functional p53 [95]. This strategy focuses on activating a tumor suppressor instead of inhibiting an oncogenic driver. Genetic studies support the physiological relevance of the MDM2–p53 autoregulatory feedback loop [96, 97]. In this loop, p53 binds to the P2 promoter of MDM2, increasing MDM2 expression and therefore increasing protein levels. MDM2 then inhibits the p53-mediated transcription of MDM2 and other downstream target genes by binding to p53, blocking its transactivation domain. Through E3 ubiquitin ligase activity, MDM2 promotes ubiquitination of p53, leading to increased p53 degradation. In one such study, MDM2 overexpression at an early stage of differentiation resulted in neutralization of p53 tumor suppressor function and a predisposition to tumorigenesis [95, 97]. Thus, inhibition of this interaction is an important focus of scientific research and drug development. In hematologic malignancies, in which TP53 is infrequently mutated, targeting MDM2 is a particularly attractive therapeutic strategy [98]. MDM2 inhibition is also being assessed with solid tumors, with some currently being investigated in phase 1 trial [99, 100]. As monotherapy, MDM2 inhibitors have generally exhibited modest clinical responses. Although preclinical evidence of MDM2 inhibitors as monotherapy is abundant, however, several combination studies are underway in clinical testing [95]. On a similar note, our study proposed a new therapeutic strategy of alone or co-inhibition of AKT1 and MDM2 with repurposed drugs for preventing E2F1-driven metastatic progression.

We also looked into the compensatory opposing paths that could result in drug resistance. In the context of our significant molecules (AKT1 and MDM2), a few paths leading to the EMT were found. For example, Inhibiting AKT1 will cause a decrease in Bcl2 expression, which will then induce p53 and MDM2 levels, causing in loss of CDH1 which in turn triggers EMT. Nevertheless, this path won’t have a compensating effect on the EMT because MDM2 inhibition is also being carried out. Likewise, alternative paths from AKT1, consisting of BLC2 and FOXO3 molecules, lead through to the EMT via MDM2. As BLC2 and FOXO3 only regulate p53 within the network, their effects are cancelled by MDM2 through a negative feedback loop to p53, preventing the induction of EMT. The simulation results also confirm that the inhibition of AKT1 or MDM2 can downregulate the EMT phenotype and we checked this propagation throughout the core network. It demonstrates that we can still achieve the effect of downregulation on the EMT phenotype in the presence of compensatory pathways. Therefore, it is proposed that a reduction in the EMT phenotype, by performing combinatorial drug inhibition on AKT1 and MDM2 cannot be bypassed by compensatory pathways.

Repurposed drugs have shown promise in clinical trials for cancer, while some fail to meet expectations [101]. Hence, while considering drug repurposing for cancer therapy, it is crucial to carefully assess the limitations. The repurposed drugs should be more susceptible to drug resistance, not exhibit unexpected toxicity, and not show severe side effects when used to treat cancer. Tadalafil, that is being considered for repurposing against AKT1 in present study, belongs to a class of Phosphodiesterase-5 (PDE5) inhibitors [57]. Due to the following effects, repurposing PDE5 inhibitors for possible use in cancer treatment has generated some research interest: (i) It has been demonstrated that PDE5 inhibitors, like sildenafil, affect blood vessel dilatation and blood flow. This may be used to increase the blood flow to tumors, which would facilitate the cancer treatments like chemotherapy to reach the tumor [102]. (ii) PDE5 inhibitors have anti-angiogenic properties. They may slow the growth of cancer by preventing angiogenesis [103]. (ii) PDE5 inhibitors have immunomodulatory effects. Modulating immune system may improve the body’s ability to identify and attack cancer cells, which makes it a valuable therapeutic option for cancer patients [104]. (iii) Tadalafil may prevent cancer cell invasion and metastasis, potentially through altering cell adhesion mechanisms according to some preliminary data [57]. Because of these plausible mechanisms, Tadalafil is an important drug in cancer research; however, to fully understand the advantages and disadvantages of repurposing Tadalafil for cancer, preclinical studies and clinical trials are required.

Finasteride is another drug that has been suggested for repurposing against MDM2. Although Finasteride is commonly used to treat cancer, some research has looked into its possible application in the prevention and management of prostate cancer [70]. Finasteride was found to lower participants’ risk of prostate cancer by about 25% in the Prostate Cancer Prevention Trial (PCPT) [105]. This means that 5-alpha reductase inhibitors, such as finasteride, may be used to prevent prostate cancer especially in people who are at high risk. In some cases, finasteride used in combination with other therapies for prostate cancer. Although, it is not the main component of treatment, but it can be considered as a part of a comprehensive treatment plan. Finasteride inhibits the growth of prostate cancer cells by lowering the body’s dihydrotestosterone levels [106]. Other 5-alpha reductase inhibitors, such as dutasteride, is also being investigated for the prevention and treatment of prostate cancer [71].

Clinical evaluation of the advantages and disadvantages of repurposing drugs for cancer treatment is crucial to gain a substantial benefit as there are also several limitations associated with the computational workflow.

(i) One of the limitations in the development of a computational workflow poses by the integration of data from different databases and tools. Since they each have different standard formats and applies different strategy to create assumptions. To overcome this limitation, we used proper annotations to remove inconsistencies that exist and made effective integration of data in the E2F1 network map.
(ii) Another concern is related to carrying out the inferences from the network map. Since, the actual number of theoretically possible interactions between network components (miRNAs, marker proteins, receptor proteins and their target proteins, etc.) far exceeds the number of true interactions that can be biologically true. There is no single best method exists and various methods feature complimentary interaction types. Hence, in future studies, ensemble approaches that aggregate the outcomes of several methods, will improve the accuracy of the predicted interactions. To overcome this limitation, we have made an online portal to access the E2F1 map (https://navicell.curie.fr/pages/maps_e2f1.html) and available for data analysis, navigation, and curation by users.
(iii) Although to cross-validate our molecular docking and screening findings, we ran MD simulations that confirmed the stability of the compound drugs identified for protein signatures. We acknowledge that further laboratory and clinical studies are needed to validate the inhibitory effects of these FDA-approved drugs against AKT1 and MDM2 as potential drugs for melanoma cancer.

## Conclusions

Cancer is a disease where multiple pathways are dysregulated, and its development and progression involve both independent and overlapping molecular targets. Advanced computational methods can unravel the properties of cancer-related proteins and their interactions in the molecular networks and enable the designing of next-generation targeted therapeutics. With the computational pipeline used in this study, we were successful in the identification of key protein signatures (AKT1 and MDM2) in melanoma from a core regulatory network that is based on a published E2F1 interaction map. In this work, a hybrid approach of logic-based modeling coupled with computer-aided drug design techniques was applied for the identification of drug candidates that can modulate the protein’s activity and could be possibly used for melanoma research. From virtual screening, the top candidate drugs based on the lowest binding affinity values against protein signatures were reported and evaluated for their safety profiles. MD simulations confirmed the stability of the two candidate drugs (Tadalafil and Finasteride) in complex with protein signatures over the course of 100 ns trajectory analysis. In conclusion, Tadalafil and Finasteride were predicted to be potent drugs to target AKT1 and MDM2 respectively; and may increase cell death in melanoma cancer cells, and this effect is mediated in the presence of E2F1. These findings would facilitate the development of effective inhibitors for clinical use in melanoma metastasis.

## Methods

### Network analysis and motif identification

The Cytoscape version of the E2F1 map was downloaded from https://sourceforge.net/projects/e2f1map/files and converted into a format suitable for the Cytoscape plugin NetDS v3.0 [18]. The purpose of this was to identify important nodes and network motifs in the network. The loop length was set to three nodes and feedback motifs (n = 444) were retrieved. We then used the Cytoscape plugin NetworkAnalyzer to evaluate the topological properties of nodes [107]. More specifically, we calculated the average number of neighbors for each node in the network (degree) [108] and the density of connections among the neighbors of a node (betweenness centrality) [109] to understand the overall organization of the network. Among non-topological properties, we calculated the number of nodes in a motif involved in the KEGG melanoma pathway (KEGG: 05218), and a prioritization score for each gene from the web resource DISEASES [110].

### Array data from aggressive melanoma cell lines

We used gene expression data from a previous study generated in SK-Mel103 and SK-Mel-147 cell lines (obtained from Dr. M. Soengas) with and without endogenous E2F1 depletion as described [3].

### Motif prioritization

The regulatory motifs were prioritized using a ranking score for each motif considering key topological and non-topological properties with respect to the relevance of the melanoma phenotype. The motif ranking score is calculated using Eq. (1).

1 $${Ranking score}_{ij}=\frac{{W}_{1j}}{2} \left(\frac{(ND)i}{\text{max }(ND)} + \frac{(BC)i}{\text{max} (BC)}\right)+ {W}_{2j} \frac{(DP)i}{\text{max }(DP)}+ {W}_{3j} \frac{(GP)i}{\text{max }(GP)}+ {W}_{4j} \frac{(|FC|)i}{\text{max }(|FC|)}$$

The equation uses a multi-objective function which is normalized to the maximum property value under consideration. We used a ranking scheme that was previously developed [10] by assigning different weights to various topological and non-topological parameters. In particular, the weights to two topological parameters (node degree ⟨ND⟩ and betweenness centrality ⟨BC⟩) were divided into half to avoid overemphasis on the topological properties and assigning equal weighting factors W2j-W4j to give equal importance to other properties (disease pathway association ⟨DP⟩, gene prioritization score ⟨GP⟩, Log2 fold change ⟨|FC|⟩) in motif prioritization. The equation generates a ranking score for each motif i (1…n) depending on the sets of values chosen for the weighting scenarios j (1 to 13) shown in additional supp files 1a-c. Later, the top 10 motifs were selected from each of the weighting scenarios (13*10 = 130 motifs). Furthermore, a unique set of motifs were identified and processed for the construction of a melanoma-specific core regulatory network. The optimization of multi-objective function is discussed in detail [10].

### Derivation of a core regulatory network

All the top-ranked motifs identified in the previous steps were merged to create a regulatory core. Additionally, we also considered receptor proteins as critical factors determining the EMT phenotype and directly interacting/ regulating nodes present in the top-ranked motifs. In total, we found and included ten receptor proteins (AR, ESR1, FGFR1, FLT4, NR2F2, NR4A1, TGFBR1, TGFBR2, THRA, and THRB) into the regulatory core. These receptor proteins are the first neighbors of ranked motif nodes and are present in the E2F1 map. In addition, we added four EMT marker proteins (CDH1, VIM, ZEB1, and SNAI1) and direct connections with motif nodes (additional supp file 1d) in our regulatory core. The rationale behind selecting the specific nodes (ZEB1, CDH1, VIM, SNAI1) is due to the fact that these are known players in EMT which is characterized by a loss (downregulation) of epithelial cell marker CDH1, followed by an upregulation in the expression of mesenchymal cell markers such as VIM, SNAI1 and ZEB1 in primary tumors [39, 40]. The motivation was to determine the EMT process as a driver of invasive phenotype in melanoma by using a logic function involving these EMT markers.

### Logic-based modeling to derive protein signatures

To identify protein signatures in the regulatory core, the network is translated into a logic-based model, and in silico perturbation experiments were performed in the software tool CellNetAnalyzer (CNA) [111]. For this, we derived boolean rules for the input (receptor proteins) layer and a synchronous update scheme is used to propagate signals from the input layer to the output layer through the nodes present in the regulatory layer. We used the logical steady-state (LSS) attractor algorithm to determine the steady-state values of all the nodes at the same time step. The network is simulated to determine the impact of the input layer vectors on the EMT phenotype (output layer). We performed single and double perturbation experiments iteratively for the initial conditions that are determined through the additional publicly available gene expression dataset (GSE46517) from Gene Expression Omnibus (GEO). The perturbation experiments were performed by changing the boolean state of each node alone and in combination with other nodes in the regulatory layer to see the impact on the invasiveness (https://github.com/nivisingh22/Melonoma_core_model). Those node(s) which upon inhibition change the EMT to minimum level or upon activation to maximum level are further evaluated as effective protein signatures associated with EMT transition in melanoma.

### Virtual screening of repurposable drugs

Virtual screening was performed as follows:

#### FDA-approved drug library preparation

The FDA-approved drug library was downloaded from the ZINC12 (http://zinc.dock-ing.org/zinc/) database. Since the library contains 2D structures, Open Babel 3.1.1 (https://pypi.org/project/openbabel/3.1.1/) was used to generate 3D energy-minimized structures to be utilized for docking studies.

#### Protein structure preparation

The crystal structure of protein signatures AKT1 (PDB: 3OCB) and MDM2 (PDB: 3JZK) were downloaded from the RCSB Protein Data Bank (https://www.rcsb.org/). Proteins were pre-processed by removal of heteroatoms, adding polar hydrogens, and gasteiger charges using the AutoDock Vina [112]. Further, the coordinates of the active site residues were determined.

#### Binding affinity prediction using molecular docking

Virtual screening was carried out in PyRx v0.8 (AutoDock Vina-based) software [113]. The library compounds were first imported as SDF files in the open babel of PyRx and further energy minimization (using Universal Force Filed) of all the library compounds was performed followed by conversion into PDBQT format files. Later, a gird box was designed to cover the binding site residues within the protein signatures and then the prepared FDA-approved drug library was subjected to docking against AKT1 and MDM2. To efficiently explore the docking conformational space, the search efficiency was set at 100%. For docking calculations, 9 con-formers were generated for each ligand-protein complex. The resulting ligand-docked poses were compared with the crystallographic poses based on ≤ 2.0 Å RMSD tolerance on the heavy atoms. The best predicted binding mode and the corresponding binding affinity (or binding free energy) in kcal/mol were selected (Fig. 1D). The more negative numerical values of binding free energy represent the better binding between a ligand and a protein signature. The docked complexes and graphical visualization were done in DS Visualizer [114].

#### Safety profile assessment of candidate drugs

The candidate drugs that bind to the protein signatures (in total, 1254 with AKT1 and 1257 with MDM2 respectively) were subjected to safety profile assessment based on ADMET risk, pharmacokinetics, drug-likeness, and medicinal chemistry friendliness prediction (Fig. 1D) using the SwissADME tool (http://www.swissadme.ch/) [115]. The detailed properties/score values of the candidate drugs are provided (in the additional supp file 2a) along with the binding affinities (in kcal/mol) for both protein signatures separately (additional supp file 2b-c).

#### Molecular dynamics simulation (MDS)

Schrödinger LLC Desmond software was used to simulate the docked complexes for 100 nanoseconds (Fig. 1D) [116]. For this, the complexes were pre-processed in Maestro Wizard for preparation and refinement steps. Missing side chains and loops were added using Prime, optimized with ProtAssign, and minimized using the OPLS_2005 force field. Further, the System Builder tool was implemented to build a system for simulation. The system was created in an orthorhombic box and the TIP3P water solvation model was used with default boundary conditions. The system was neutralized by adding counter ions and salt concentration set to 0.15 M NaCl to mimic physiological conditions. The simulation parameters were defined as follows: simulation time 100 ns, recording interval trajectory 100 ps, ensemble class NPT, pressure bar 1.01325, and temperature 300 K. The stability of the simulation was verified by comparing the protein and ligand RMSD over time. The interactions between the protein and ligand were analyzed in the Desmond simulation interaction diagram tool.

### Electronic supplementary material

Below is the link to the electronic supplementary material.

**Supplementary Material 1:** Identification and prioritization of motifs to derive a core regulatory network: (1a) FBLs in E2F1 map and ranking score (1b) top-ranked FBLs in melanoma (1c) weighting scenarios for motif prioritization and (1d) regulatory core interactions

**Supplementary Material 2:** Safety profile assessment of candidate drugs and prediction of binding affinity: (2a) Prediction of ADMET risk, pharmacokinetics, drug-likeness, and medicinal friendliness score of FDA-approved candidate drugs docked to protein signatures AKT1 and MDM2. (2b) binding affinity of AKT1 to candidate drugs and (2c) binding affinity of MDM2 to candidate drugs

**Supplementary Material 3:** Network analysis methodology

## Data Availability

All datasets generated for this study are included in the article/additional supplementary information. The boolean model is available to download in CellNetAnalyzer format with simulation/input files, and MATLAB code for automated simulation of input scenarios at https://github.com/nivisingh22/Melonoma_core_model.

## Abbreviations

GRNs: gene regulatory networks
EMT: epithelial-mesenchymal transition
FDA: food and drug administration
CADD: computer-aided drug design
ADMET: absorption, distribution, metabolism, excretion and toxicity
PL RMSD: protein-ligand root mean square deviation
P RMSF: protein root mean square fluctuation
SSE: secondary structure elements
TPSA: topological polar surface area
MW: molecular weight

## Description of gene symbols appearing in the network

AKT1: AKT serine/threonine kinase 1
AR: androgen receptor
AXIN2: axin 2
BCL2: B-cell lymphoma-2
CCNA2: cyclin A2
CCNB1: cyclin B1
CCNE2: cyclin E2
CDC20: cell division cycle 20
CDH1: cadherin 1
CDK1: cyclin dependent kinase 1
CDK2: cyclin dependent kinase 2
CDKN2A: cyclin dependent kinase inhibitor 2A
CTNNB1: catenin beta 1
E2F1: E2F transcription factor 1
E2F2: E2F transcription factor 2
E2F3: E2F transcription factor 3
ESR1: estrogen receptor 1
FGFR1: fibroblast growth factor receptor 1
FLT4: fms related receptor tyrosine kinase 4
FOXO3: forkhead box O3
KIAA1524: cancerous Inhibitor Of Protein Phosphatase 2A
LEF1: lymphoid enhancer binding factor 1
MDM2: Mouse double minute 2 homolog
MDM4: Mouse double minute 4 homolog
MYC: Myelocytomatosis
NR2F2: nuclear receptor subfamily 2 group F member 2
NR4A1: nuclear receptor subfamily 4 group A member 1
PPP2R1A: protein phosphatase 2 scaffold subunit A alpha
PPP2R1B: protein phosphatase 2 scaffold subunit A beta
PPP2R2A: protein phosphatase 2 regulatory subunit B alpha
PPP2R2D: protein phosphatase 2 regulatory subunit B delta
PI3K: phosphatidylinositol 3-kinase
PIP3: phosphatidylinositol-3,4,5-triphosphate
RB1: RB transcriptional corepressor 1
SIRT1: sirtuin 1
SNAI1: snail family transcriptional repressor 1
SP1: Sp1 transcription factor
TGFBR1: transforming growth factor beta receptor 1
TGFBR2: transforming growth factor beta receptor 2
THRA: thyroid hormone receptor alpha
THRB: thyroid hormone receptor beta
TP53: tumor protein p53
VIM: vimentin
ZEB1: zinc finger E-box binding homeobox 1
