# Supplementary material for: Logic-based modeling and drug repurposing for the prediction of novel therapeutic targets and combination regimens against E2F1-driven melanoma progression
Source: BMC Chem. 2023 Nov 22;17(1):161. doi: 10.1186/s13065-023-01082-2 (PMC10666365; doi:10.1186/s13065-023-01082-2)
Supplement: Supplementary file 3 — Supplementary Material 3: Network analysis methodology [file 13065_2023_1082_MOESM3_ESM.pdf]

Network analysis methodology

Topological parameters used in the E2F1 network analysis:

To evaluate the structural properties of the E2F1 network, we converted all types of regulatory interactions including activation, inhibition, and neutral from source to target (Supp additional file 1d) into Cytoscape suitable format. Using the NetworkAnalyzer plugin, we calculated all the parameters of the E2F1 network (Supp Table 1).

Supp Table 1: Statistics of interactions in the E2F1 network.

Number of nodes	1015	Network diameter	8
Number of edges	4179	Network radius	4
Network density	0.008	Average clustering coefficient	0.226
Isolated nodes	0	Average characteristic path length	3.257
Self-loops	33	Average no of neighbors	7.894

The E2F1 network has 1015 nodes and 4174 edges. The average number of neighbors for each node in the network is 7.894 indicates that the network is well-connected (isolated nodes are 0). The average clustering coefficient of the network is 0.226 and a large network diameter of 8 shows the modular organization of nodes in the network [1,2]. Further, a large average characteristic path length and a small average clustering coefficient suggest that the network has a small-world architecture and the small-world property reveals that signals can propagate very fast through the whole network [2,3,4].

The topological properties (i) node degree and (ii) betweenness centrality of the network were mapped for visualization. The node degree distribution (k) gives the number of neighbors for each node (n) *i.e.* the number of edges linked to a node in the network (Supp Fig 1). A power law (red line) $y = ax^b$ was fitted where y indicates the number of nodes that share a particular degree at x (a = 323.74 and b = -1.274, R-squared = 0.842, correlation = 0.850). Genes with high degrees are in the right-down region of the graph. This leads us to the conclusion that the network has a scale-free topology, which is compatible with the network's sparse presence of a few high-degree nodes, called hubs, such as E2F1 (degree = 421). Such networks that contain a few hubs are generally heterogeneous in terms of node degree and are seen as being robust to single random perturbations [2,5].

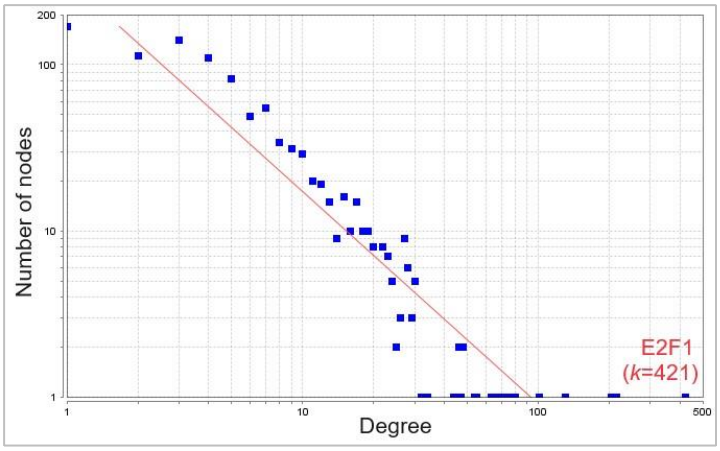

Supp Fig 1: Node degree distribution of the network.

In Supp Fig 2, the betweenness centrality (C*b*) measures the density of connections among the neighbors of a node (n) *i.e.* the amount of control that a particular node exerts over the interactions of the other nodes in the network [6]. A power law (red line) $y = ax^b$ was fitted where y indicates the betweenness value for each node vs the number of neighbor nodes at x (a = 0.0 and b = 2.108, R-squared = 0.477, correlation = 0.953). Genes with high betweenness values are in the right=up region of the graph. From this, we conclude that E2F1, followed by E2F2 and E2F3, has very high node degrees and high betweenness values. This is understandable because the network was built by focusing on interactions around the E2F family. Other nodes with high node degrees are p53 [7] and MYC [8], are known for their role in melanoma tumorigenesis. Particularly, E2F1 has the greatest betweenness value of 0.423, indicating that it is crucial for the network's signal flow.

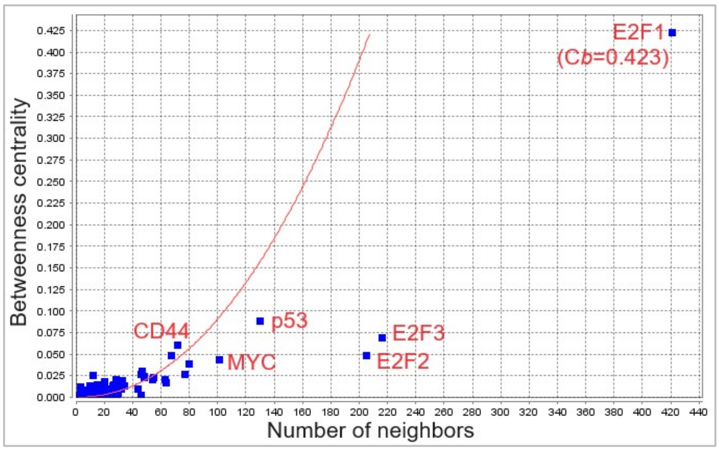

Supp Fig 2: Betweenness centrality distribution of the network.

<u>Robustness of E2F1 network:</u>

We calculated the robustness of the E2F1 network using MORO, Cytoscape app [9] and compared it with the two existing large-scale signaling networks- the canonical cell signaling network (http://stke.sciencemag.org) and the human signal transduction network (<u>http://www.bri.nrc.ca/wang</u>). Directed networks are loaded for the analysis, the parameters are chosen (Update-Rule Scheme CONJ-DISJ and No. of initial states:10), and batch mode simulation is performed to calculate the robustness of the networks. Supp Table 2 displays the analysis results. E2F1 network's sRobustness (robustness against initial-state perturbation) and rRobustness (robustness against update-rule perturbation) values were found to be fairly close to the trend of the other two stable networks. It has been also suggested that a modular organization of cancer signaling networks is associated with patient survivability which suggests a relationship between modularity and network robustness [10]. High modularity values (>0.5) indicate that the networks have dense connectivity between the nodes.

Supp Table 2: Robustness analysis of the networks.

Analysis parameters	Canonical cell signaling network	Human signal transduction network	E2F1 network
Nodes	754	5443	1015
Edges	1624	37663	4179
sRobustness	0.67721	0.754	0.68966
rRobustness	0.77521	0.6524	0.69143
Modularity	0.72825	0.54534	0.51674

Regulatory network motifs:

The E2F1 network is of recurring structural patterns called network motifs which are used by a cell to process information and to govern dynamic response to external or internal fluctuations [11-13]. Such network motifs include feedback and feedforward loops (FBLs and FFLs) that are present among various regulatory network layers and are commonly encountered in cancer networks [14,15]. FBLs are characterized by the direct or indirect inhibition or activation of a node by its own target, and depending on the parity of the negative linkages in the loop, they can be positive or negative (Supp Fig 3) [16]. However, FFLs are composed of an input node that controls an intermediate node, while the two also control an output node. Depending on the parity of the loop's negative linkages, the FFLs can either be coherent or incoherent [16]. Both genes that code for proteins and those that do not are accompanied by such regulatory motifs [17].

	Architecture (examples)	Condition (EMT activation)
A (i)	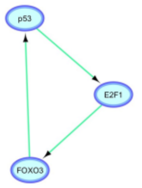	As FOXO3 becomes active, it leads to stabilization of the p53, and in turn E2F1 acts upstream of p53, and positively affects FOXO3 protein levels resulting in the formation of a positive feedback loop.
B (ii)	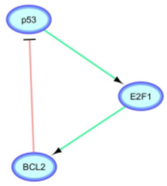	BLC2 inhibits p53 transcriptional activity, and p53 deficiency causes E2F1 reduction and, later E2F1 loss results in decreased BCL2 expression forming a negative feedback loop.
(iii)	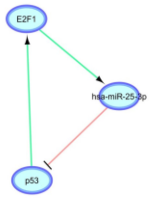	Transcriptional activation of p53 results in increased E2F1 activity, in turn has-miR-25-3p activated by E2F1. Later, induction of hsa-miR-25-3p negatively regulates p53 levels.

Supp Fig 3: Examples of A(i) positive feedback loop and B(ii, iii) negative feedback loops from melanoma core network.

Such gene sets are highly interconnected, involved in specific phenotypes, and regulate one another through regulatory loops (motifs) from various pathways and the analysis of these can provide insights into the structure and dynamics of the network followed by the identification of therapeutic targets [18]. These targets can be challenging to identify without the assistance of computational analysis due to the large and complex quantitative data, which requires computational algorithms for the analysis [19]; as well as the need to take into account a number of factors such as target-related safety issues, druggability, assayability, disease mechanisms, reduction in animal testing, time and cost considerations [20]. Vera and coauthors, outlined the necessity of computational strategies in melanoma diagnostics and therapy [19].

Supplementary References:

1. Ravasz E, Somera AL, Mongru DA, Oltvai ZN, Barabási AL. Hierarchical organization of modularity in metabolic networks. science. 2002 Aug 30;297(5586):1551-5.

2. Barabasi AL, Oltvai ZN. Network biology: understanding the cell's functional organization. Nature reviews genetics. 2004 Feb 1;5(2):101-13.

3. Watts DJ, Strogatz SH. Collective dynamics of ‘small-world ‘networks. nature. 1998 Jun;393(6684):440-2.

4. Fell DA, Wagner A. The small world of metabolism. Nature biotechnology. 2000 Nov;18(11):1121-2.

5. Albert R, Jeong H, Barabási AL. Error and attack tolerance of complex networks. nature. 2000 Jul 27;406(6794):378-82.

6. Abbasi A, Hossain L, Leydesdorff L. Betweenness centrality as a driver of preferential attachment in the evolution of research collaboration networks. Journal of informetrics. 2012 Jul 1;6(3):403-12.

7. Boutelle AM, Attardi LD. p53 and tumor suppression: it takes a network. Trends in cell biology. 2021 Apr 1;31(4):298-310.

8. Urbanski L, Brugiolo M, Park S, Angarola BL, Leclair NK, Yurieva M, Palmer P, Sahu SK, Anczuków O. MYC regulates a pan-cancer network of co-expressed oncogenic splicing factors. Cell reports. 2022 Nov 22;41(8).

9. Truong CD, Tran TD, Kwon YK. MORO: a Cytoscape app for relationship analysis between modularity and robustness in large-scale biological networks. BMC Systems Biology. 2016 Dec;10:521-30.

10. Takemoto K, Kihara K. Modular organization of cancer signaling networks is associated with patient survivability. Biosystems. 2013 Sep 1;113(3):149-54.

11. Knoll S, Fürst K, Kowtharapu B, Schmitz U, Marquardt S, Wolkenhauer O, Martin H, Pützer BM. E2F1 induces miR-224/452 expression to drive EMT through TXNIP downregulation. EMBO reports. 2014 Dec;15(12):1315-29.

12. Goody D, Gupta SK, Engelmann D, Spitschak A, Marquardt S, Mikkat S, Meier C, Hauser C, Gundlach JP, Egberts JH, Martin H. Drug repositioning inferred from E2F1-Coregulator interactions studies for the prevention and treatment of metastatic cancers. Theranostics. 2019;9(5):1490.

13. Singh N, Eberhardt M, Wolkenhauer O, Vera J, Gupta SK. An integrative network-driven pipeline for systematic identification of lncRNA-associated regulatory network motifs in metastatic melanoma. BMC bioinformatics. 2020 Dec;21(1):1-7.

14. Khan FM, Sadeghi M, Gupta SK, Wolkenhauer O. A network-based integrative workflow to unravel mechanisms underlying disease progression. Systems Biology. 2018:247-76.

15. Le DH, Kwon YK. NetDS: a Cytoscape plugin to analyze the robustness of dynamics and feedforward/feedback loop structures of biological networks. Bioinformatics. 2011 Oct 1;27(19):2767-8.

16. Khan FM, Sadeghi M, Gupta SK, Wolkenhauer O. A network-based integrative workflow to unravel mechanisms underlying disease progression. Systems Biology. 2018:247-76.

17. de Azevedo AL, Carvalho TM, Mara CS, Giner IS, de Oliveira JC, Gradia DF, Cavalli IJ, Ribeiro EM. Major regulators of the multi-step metastatic process are potential therapeutic targets for breast cancer management. Functional & Integrative Genomics. 2023 Jun;23(2):171.

18. Liao J, Wang Q, Wu F, Huang Z. In silico methods for identification of potential active sites of therapeutic targets. Molecules. 2022 Oct 20;27(20):7103.

19. Vera J, Lai X, Baur A, Erdmann M, Gupta S, Guttà C, Heinzerling L, Heppt MV, Kazmierczak PM, Kunz M, Lischer C. Melanoma 2.0. Skin cancer as a paradigm for emerging diagnostic technologies, computational modelling and artificial intelligence. Briefings in Bioinformatics. 2022 Nov;23(6):bbac433.

20. Emmerich CH, Gamboa LM, Hofmann MC, Bonin-Andresen M, Arbach O, Schendel P, Gerlach B, Hempel K, Bespalov A, Dirnagl U, Parnham MJ. Improving target assessment in biomedical research: the GOT-IT recommendations. Nature reviews Drug discovery. 2021 Jan;20(1):64-81.
